# Game-theoretical model for the sustainable use of thermal water resources: the case of Ischia volcanic Island (Italy)

**DOI:** 10.1007/s10653-021-00871-9

**Published:** 2021-04-12

**Authors:** Richárd Kicsiny, Vincenzo Piscopo, Antonino Scarelli, Zoltán Varga

**Affiliations:** 1 Department of Mathematics and Modelling, Institute of Mathematics and Basic Science, Hungarian University of Agriculture and Life Sciences, Godollo, Hungary; 2 Department of Ecological and Biological Sciences, Tuscia University, Viterbo, Italy

**Keywords:** Thermal water, Sustainable yield, Game-theoretical modeling, Island of Ischia

## Abstract

The Island of Ischia, one of the Italian active volcanoes, is a famous tourist resort for spa treatments. Spas are supplied by withdrawals from groundwaters which are characterized by a wide range of chemical compositions, salinity and temperature. In natural conditions, the hydrogeological system is recharged by rainfall and by deep fluids; the discharge is towards the sea and the springs. During the peak of the tourist season, when approximately 240 wells are operating simultaneously, a significant additional recharge of the aquifers derives from seawater and from upwelling increase in deep fluids. Although this does not compromise the availability of groundwater, the pumping often determines variation in composition and temperature of groundwater over time. Conversely, the maintenance of a stable quality of thermal waters represents one of the requirements for their therapeutic use in the spas. The study aims to establish game-theoretical modeling of the optimal sustainable exploitation of the groundwater resources of the island by competing users (spas) falling in the same flow tube of the aquifer. In the game the spas are the players, the strategy of a player consists of a fixed pumping rate and daily time durations of pumping, and the player’s utility or payoff is proportional to the total quantity of withdrawn thermal water in a given time period. A special constrained Pareto optimal strategy choice is obtained, considered as a cooperative solution of the game. Pareto optimality means that there is no other strategy choice that makes one player better off without making some other player worse off.

## Introduction

Thermal waters are widely used all over the world as a resource for health, wellness and recreational tourism with important impact on global economy (Global Wellness Institute, 2017). Business related to this market depends on maintaining the quality and quantity of thermal waters. Since the thermal waters are groundwaters originated by specific circuits in the aquifers determining their chemical–physical properties, their sustainable management implies a use of these resources “in a manner that can be maintained for an indefinite time without causing unacceptable environmental, economic, or social consequences” (Alley and Leake, 2004). In the few cases reported in the literature (Atkinson & Davison, 2002; Buday et al., 2015; Fabbri et al., 2017; Piscopo et al., 2019), the assessment of the sustainable use of thermal waters has been addressed by means of groundwater flow models or analysis of the potentiometric level trend over time.

The volcanic island of Ischia represents one of the few cases in the world where there is a very high concentration of groundwater withdrawals used for health, wellness and recreational tourism. This is due to the presence of an active hydrothermal system which gives rise to a wide variety of groundwaters, very different in chemical composition (from calcium-bicarbonate to alkali-chloride waters), salinity (from 1 to 42 g/L) and temperature (from 13 to 90 °C). 264 groundwater tapping points (244 wells and 20 springs) are distributed over an area of about 20 km^2^, mainly near the coast, and supply spas and tourism facilities. In natural conditions, the hydrogeological system of the island is mainly recharged by rainfall and by deep fluids. The pumping from the numerous wells present in the coastal area significantly increases the recharge of the island with seawater intrusion and upwelling of deep fluids. Although this does not compromise the availability of groundwater in quantitative terms, the pumping modulates the quality of the water captured by the wells often determining variation in composition and temperature of groundwater over time. A qualitative decay of the thermal waters can have a relevant economic impact, given that the European and Italian legislations establish that composition and temperature of thermal waters used for therapeutic purposes must remain constant over time (Piscopo et al., 2020a).

The purpose of this research is to evaluate the sustainable yield of the wells that supply the various spas, maximizing the profit of the spas and limiting the qualitative decline of the thermal waters. To achieve this goal, the hydrogeology of the system, the aquifer response to the pumping and the distribution of groundwater withdrawals were considered, in order to develop a game-theoretical model.

Game-theoretical modeling is a widely used approach to optimization problems in conflict situations. For the general methodology and the theoretical background of game theory we refer to the monographs of von Neumann and Morgenstern (1944) (a classical one) and Mazalov (2014) (a more recent one, with a variety of application fields). Game-theoretical approach to water resource management goes back to several decades (e.g., Bogárdi & Szidarovszky, 1976). Examples for recent developments are Kicsiny et al. (2014a), where a dynamic a Stackelberg game model was applied to water rationalization in drought emergency; in Kicsiny et al. (2014b) and Kicsiny (2017) its methodological background was worked out; in Kicsiny and Varga (2019) a differential game model with discretized solution was applied to the conflict situation concerning the time-dependent use of limited water resources.

## Study area

**Fig. 1:**
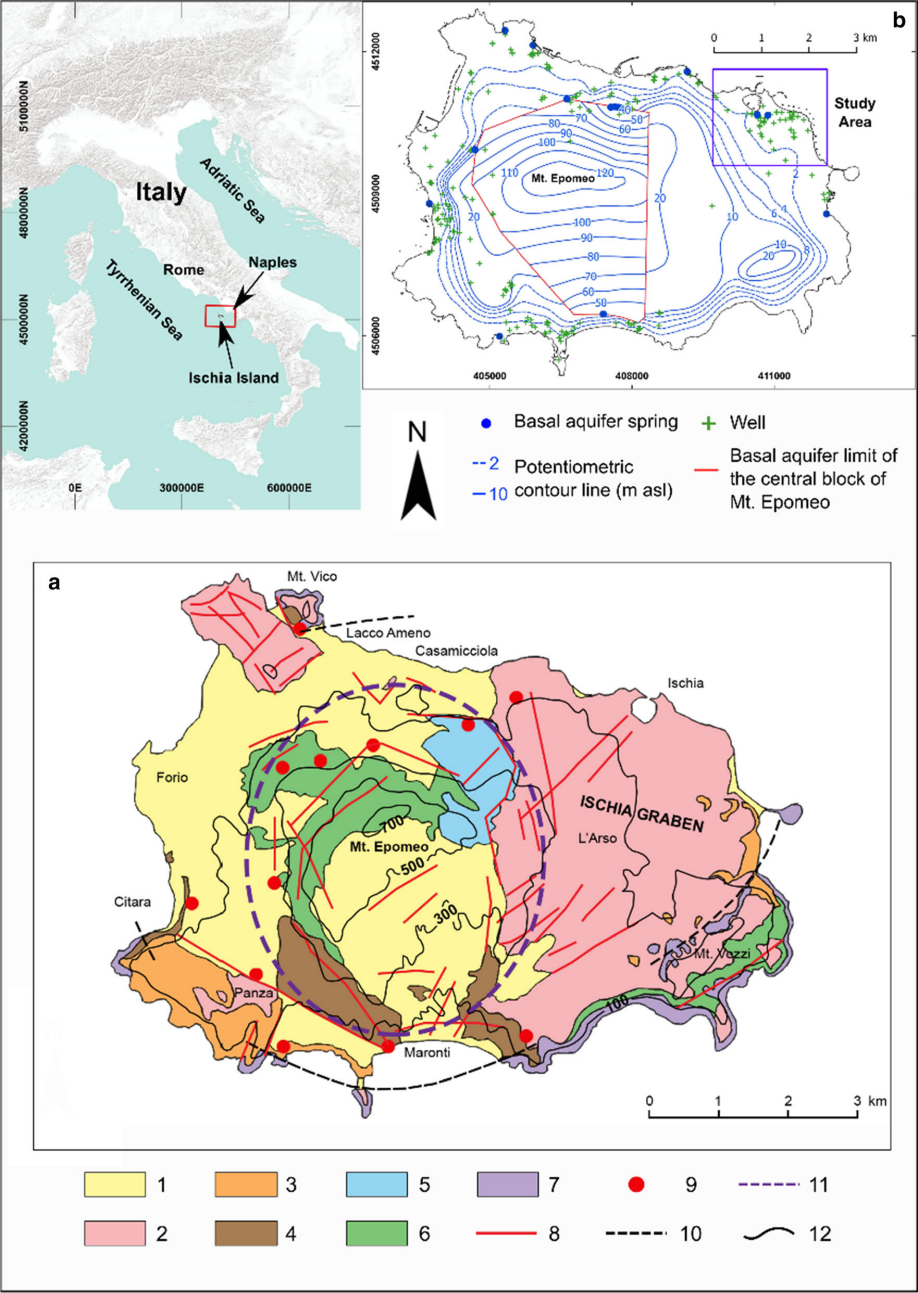
Geological and hydrogeological framework of the Island of Ischia (modified from Piscopo et al., 2020a). **a** Simplified geological map: (1) landslide and reworked pyroclastic deposits, (2) pyroclastic deposits and lavas (< 18 ka), (3) pyroclastic fall deposits, tuffs and lavas (33–18 ka), (4) tuffs and pyroclastic fall deposits (55–18 ka), (5) marine epiclastic deposits, (6) Mt. Epomeo Green Tuff (75–55 ka), (7) lavas and pyroclastic deposits (> 75 ka), (8) fault, (9) fumarole field, (10) caldera rim, (11) resurgent area, (12) elevation in m ASL. **b** Simplified hydrogeological map with the study area

The Island of Ischia is one of the active volcanoes of the Neapolitan area (Fig. 1). From 150 ka to AD 1302, volcanism of Ischia gave rise to lava flows, lava domes and pyroclastic deposits, ranging in composition mainly from trachyte to phonolite, epiclastic deposits, marine sediments and landslide deposits. The most remarkable eruption was the Mt. Epomeo Green Tuff eruption (55 ka) accompanied by the caldera collapse, followed by a resurgence phenomenon producing an uplift of the Mt. Epomeo block over time (Sbrana & Toccaceli, 2011; Vezzoli, 1988) (Fig. 1).

The volcanic island is characterized by an active geothermal system linked to the presence of a shallow magmatic body, whose surface manifestations are hot waters, fumaroles and steaming grounds. Geochemical studies conducted on the waters and fumaroles of the island have highlighted the presence of a complex hydrothermal system resulting from overlapping and interconnected reservoirs fed by meteoric waters, seawater and magma-derived or mixed magmatic-crustal gases (e.g., De Gennaro et al., 1984; Di Napoli et al., 2009; Panichi et al., 1992).

The groundwater flow in the first 200 m of depth, that is the depth crossed by the wells of the spas, is strongly conditioned by the volcano-tectonic structure of the island. Piscopo et al. (2020a) recognize an independent and uplifted basal groundwater circulation in the resurgent block of Mt. Epomeo (Fig. 1), where the marginal faults bordering the resurgent block constitute partial barriers to the basal groundwater flow and preferential ways of ascending deep fluids. Groundwater flow in the peripheral area of Mt. Epomeo is mainly influenced by the nature of the aquifer formations: in the northern, western and southern areas, a continuous basal aquifer and local discontinuous perched aquifers can be distinguished given the succession of tuffs, ignimbrites and epiclastic deposits; in the north-eastern area, characterized by the most recent volcanic deposits of the island, a single and continuous basal aquifer with the highest permeability in the island results (Fig. 1). In natural conditions, the hydrogeological system is recharged by rainfall (250–290 L/s) and by deep fluids (at least 90 L/s); the discharge is towards the sea and the springs. In the peripheral areas of Mt. Epomeo, when a total discharge of about 600 L/s is pumped simultaneously during the peak tourist season for the supply of spas, a significant additional recharge of the aquifers derives from seawater and from upwelling increase in deep fluids (Piscopo al., 2020a).

The quality of groundwater extracted from the wells depends not only on natural phenomena (meteoric recharge, seawater intrusion and rising of deep hydrothermal fluids), but also relies on the island sector where groundwater is pumped and on the pumping method. The distance of the wells from the coast, the well depth, the local transmissivity of the aquifer, the operating flow rate of the wells, the achievement or not of a steady-state drawdown condition during pumping are the main factors that determine variation in composition and temperature of groundwater over time (Piscopo et al., 2020b). Specifically, the waters extracted from the wells located near the coast are influenced by seawater, depending on the local elevation of the groundwater level and on the drawdown induced by pumping. For wells located far from the coast, where the seawater interface is deeper as a consequence of the higher elevation of the groundwater level, an increase in upwelling of deep hydrothermal fluids during pumping can occur, depending on the drawdown induced by pumping. In both cases, variations in the composition and temperature of the water extracted from the wells over time were verified (Piscopo et al., 2020b).

## Materials and methods

### Hydrogeological constraints for the use of thermal waters

One of the sectors of the island with the highest density of groundwater withdrawal is the north-eastern area (Fig. 1). Under undisturbed conditions (i.e., without pumping from wells), a natural groundwater flow of approximately 0.05 L/s per unit length of coastline occurs from inland towards the sea. In the pumping period (i.e., in the operating season of spas), an increase in inflow from the upstream boundary of 14% occurs during the pumping and 19% of the water pumped from the wells derives from seawater (Piscopo et al., 2020a). From the analysis of the water extracted from certain wells monitored in the period 2006–2019, it appears that the composition and temperature of the water vary over time mainly as a function of the pumping flow rate, the proximity of the wells to the coast line, the local transmissivity of the aquifer and the elevation of the potentiometric level under undisturbed conditions (Piscopo et al., 2020b).

**Fig. 2:**
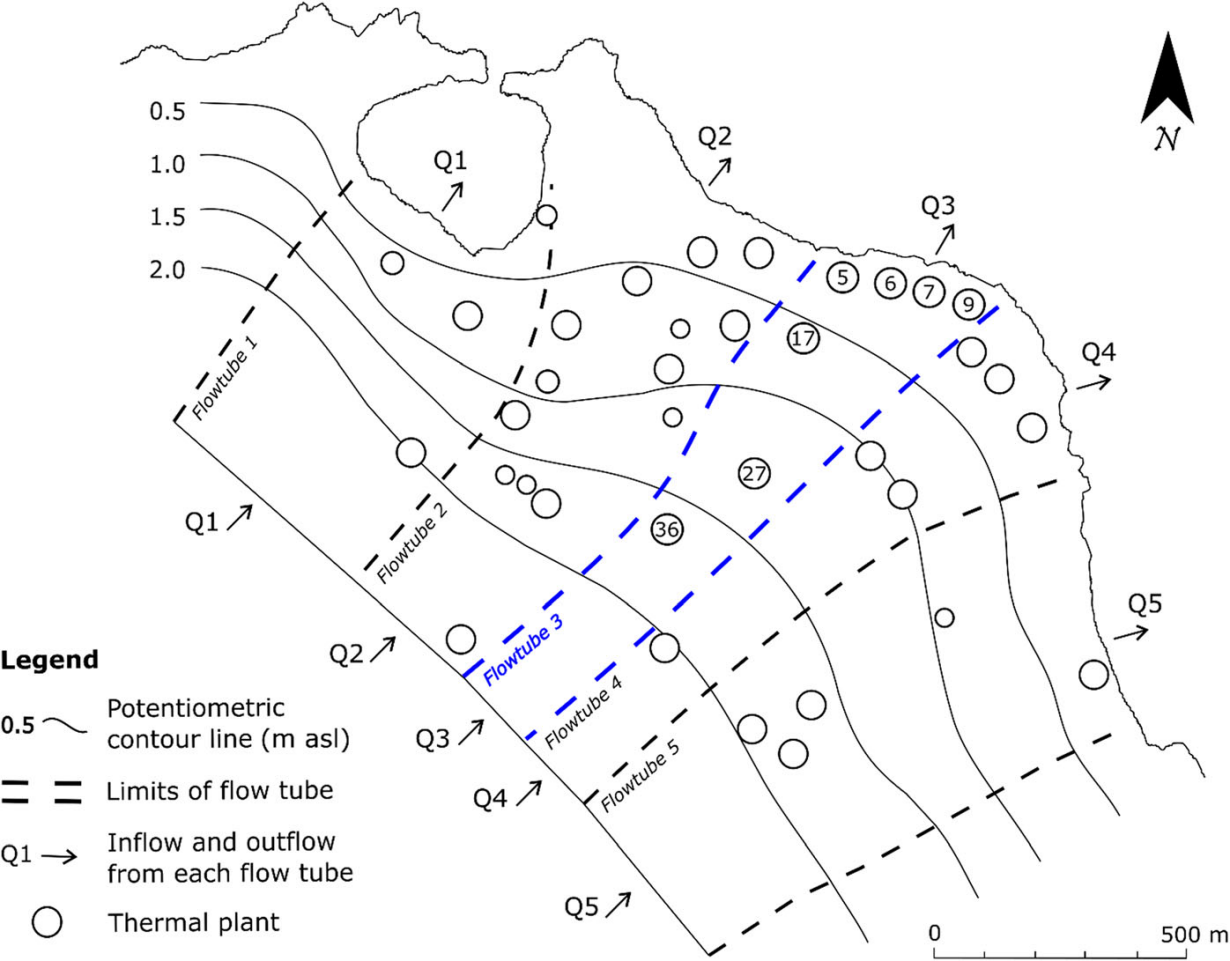
North-eastern area of the island under consideration showing the different plants within the distinguished flow tubes; the flow tube 3 was selected for the mathematical model

The north-eastern area of the island (Fig. 2) has been selected to assess the sustainability of groundwater withdrawals with the following objectives: (a) containing the qualitative decline of the waters supplying the spas; and (b) maximizing their use.

For the spa facilities falling in the area (Fig. 2), groundwater volumes required from the different plants were estimated, distinguishing volumes needed to fill the pools with thermal waters (VP) and those required for spa treatments (VF). This distinction is necessary as the daily income for the two uses is different: VP and VF were estimated at 5 and 80 € per m^3^, respectively. During the peak of the tourist season, that lasts about 180 days, the spa treatments require the VF volume for 6 h every day (from 8 am to 2 pm), while the swimming pools can be filled in the remaining 18 h.

The various groundwater users were then located in the hydrogeological context of the area. As shown in Fig. 2, the different plants and therefore the different groundwater withdrawal centers are not homogeneously distributed. Based on the potentiometric surface contour map, reconstructed through the groundwater level measurements under undisturbed conditions, five flow tubes of the aquifer with independent groundwater flow can be identified. For each flow tube, the following characteristics have been estimated (Table 1): (i) the groundwater flow rate (*Q*) and daily volume in natural conditions (*V*), considering the average flow of the aquifer per meter of width reported in the literature (0.05 L/s per m, in Piscopo et al., 2020a); (ii) the volumes VP and VF of groundwater withdrawals expected by the users falling in the each flow tube.

**Table 1:** Main characteristics of flow tubes of the area under examination: *Q* groundwater flow rate, *V* daily volume of groundwater flow, *VP* groundwater volume required to fill the pools, *VF* daily groundwater volume required for spa treatments

Flow tube	*Q* (L/s)	*V* (m^3^/day)	VP (m^3^)	VF (m^3^)
1	24.6	2125	480	68
2	17.3	1495	840	258
3	7.3	631	1680	196
4	10.5	907	1150	137
5	20.4	1763	1040	119

In order to avoid a significant seawater intrusion and an increase in upwelling of deep fluids during pumping, which would lead to a decay of the quality of the water extracted from the wells, three constraints have been considered for each flow tube:

$$\Sigma \left( {{\text{VF}} + {\text{VP}}} \right) \le V$$

$$\Sigma Q_{{\text{w}}} \le Q$$

$$Qe_{\min } \le Q_{{\text{w}}} \le Qe_{\max }$$

where *Q*_w_ is the sustainable flow rate of the well supplying each plant, *Qe*_min_ and *Qe*_max_ are the minimum and maximum flow rate of pumping of each well. *Qe*_min_ and *Qe*_max_ depend on the local elevation of the groundwater above sea level (ASL) before pumping and its variation over time due to recharge and tides, and on the drawdown induced by pumping. To estimate *Qe*_min_ and *Qe*_max_ the following constraints can be adopted:

$$h_{{\text{p}}} \ge 0.1\,{\text{m}}$$

$$\Delta h \le 0.9\,{\text{m}}$$

where *h*_p_ is the elevation of the groundwater level ASL under pumping and Δ*h* is the drawdown induced by pumping. These constraints are addressed to contain the seawater upconing for wells that are close to the coast, and the increase in upwelling of deep hydrothermal fluids in the innermost areas of the island. To determine the values of *Qe*_min_ and *Qe*_max_, the values of the specific capacity of the wells (SC = *Q*_w_*/*Δ*h*) and the range of groundwater level ASL before pumping were considered.

As shown in Table 1, critical conditions (Σ(VF + VP) > *V*) exist for flow tubes 3 and 4.

The flow tube 3, the one with the greatest difference between required volume (Σ(VF + VP)) and available volume (*V*), was selected by way of example for mathematical modeling. On the basis of previous assumptions, the minimum (*Qe*_min_) and maximum flow rate of pumping from wells (*Qe*_max_) were determined considering the average value of the specific capacity for the sector under examination (SC = 8.2 × 10^−3^ m^2^/s), the local elevation of groundwater level ASL before pumping and its variation due to recharge and tides (Piscopo et al., 2020b). In Table 2, *Qe*_min_ and *Qe*_max_ are reported for each thermal plant, as well as the thermal water volumes requested and the daily income expected from the required volumes.

**Table 2:** Parameters of the flow tube 3

Plant	VP (m^3^)	VF (m^3^)	*Qe*_min_ (L/s)	*Qe*_max_ (L/s)	Current daily income (€)
5	260	30	0.63	2.46	3700
6	170	21	0.63	2.46	2530
7	350	41	0.63	2.46	5030
9	250	30	0.63	2.46	3650
17	250	30	0.63	6.56	3650
27	230	27	0.63	7.30	3310
36	170	17	0.63	7.30	2210

The mathematical model was set on these assumptions in order to determine the maximum profit for the thermal plants, in accordance with the aforementioned constraints.

### Mathematical model

The mathematical model was based on a game-theoretical model. Such models are appropriate to describe and solve optimization problems in conflict situations. As described in the previous section, the modeling approach can be applied to flow tubes. We note that, for the convenience of numerical calculations, we use physical units which are different from the usual units of Tables 1 and 2.

Let us suppose that in a given flow tube there are *n* units (thermal plants) considered as players in the game. Every unit operates two services: the filling of swimming pools and spa treatments.

For each unit *i* = 1,…,*n* let

- $x_{i}$ be the pumping flow rate, it should be in m^3^/h, between $Qe\min_{i} {\text{ and }}Qe\max_{i}$,
- $t_{i}$ the time duration of pumping for the spa treatments, it should be in hours, between 0 and 6 h (actually operating from 8 am to 2 pm),
- $\tau_{i}$ the time duration of pumping for filling of swimming pools, it should be in hours, i.e., between 0 and 18 h (actually operating from 2 pm to 8 am next day),
- $p_{1}$ and $p_{2}$ the economic values of 1 m^3^ of water used for spa treatments and pool, respectively.

Player *i* chooses a strategy $(x_{i} ;t_{i} ;\tau_{i} )$ from strategy set

1 $$D_{i} = [Qe\min_{i} ;Qe\max_{i} ] \times [0;6] \times [0;18]$$

The payoff (or utility) function of player *i* can be defined as the total daily income

2 $$f_{i} ((x_{1} ;t_{1} ;\tau_{1} );(x_{2} ;t_{2} ;\tau_{2} ); \ldots ;(x_{n} ;t_{n} ;\tau_{n} )) = p_{1} x_{i} t_{i} + p_{2} x_{i} \tau_{i}$$

The *n*-tuples $((x_{1} ;t_{1} ;\tau_{1} );(x_{2} ;t_{2} ;\tau_{2} ); \ldots ;(x_{n} ;t_{n} ;\tau_{n} ))$ are called *multi-strategies* of the game. It should be noted that formally all payoffs are considered as functions of all strategies, since the strategy choice of each player is limited by the following constraints:

- the total pumping flow rate for the given flow tube is limited by $Q$:
  3 $$\sum\limits_{i}^{n} {x_{i} } \le Q,$$
- and the total volume derived in one day is limited by $V$:
  4 $$\sum\limits_{i}^{n} {x_{i} } t_{i} + \sum\limits_{i}^{n} {x_{i} } \tau_{i} \le V.$$

A particular feature of this game is that the players are not free to choose a multi-strategy

$$((x_{1} ;t_{1} ;\tau_{1} );(x_{2} ;t_{2} ;\tau_{2} ); \ldots ;(x_{n} ;t_{n} ;\tau_{n} ))$$

from the Cartesian product (1), since this choice is constrained by the sustainability conditions (3) and (4). This type of game-theoretical model can also be considered as a mathematical description of a particular allocation problem of resource management (Ibaraki and Naoki, 1988).

It is at hand to introduce the set of admissible multi-strategies

5 $$G = \left\{ {z = ((x_{1} ;t_{1} ;\tau_{1} );(x_{2} ;t_{2} ;\tau_{2} ); \ldots ;(x_{n} ;t_{n} ;\tau_{n} ))\left| {(x_{i} ;t_{i} ;\tau_{i} ) \in D_{i} (i = 1, \ldots ,n),\sum\limits_{i}^{n} {x_{i} } \le Q,\sum\limits_{i}^{n} {x_{i} } t_{i} + \sum\limits_{i}^{n} {x_{i} } \tau_{i} \le V} \right.} \right\}$$

As for the solution concepts of such games, there are two main paradigms: *cooperative* and *non-cooperative* approaches. The present game-theoretical study is primarily based on the cooperative (but not coalitional) approach, so that any solution of the associated constrained vector optimization problem will be considered as a cooperative solution of the game. In fact, defining the vector-valued function

$$F = (f_{1} ;f_{2} ; \ldots ;f_{n} ),$$

let us consider the constrained vector-valued optimization problem

6 $$\begin{gathered} F(z) \to \max \hfill \\ z \in G, \hfill \\ \end{gathered}$$

that is, the restriction $F\left| {_{G} } \right.$ of function *F* to set *G*, should be optimized in multi-criterial sense, based on the concept of Pareto optimality.

#### *Pareto optimality and cooperative solution of a game*

Point z* (and also the corresponding function value *F*(z*)) is said to be *Pareto optimal*, if there is no

$$z \in G\;{\text{such}},\;{\text{that}}\;f_{i} (z{*}) \le f_{i} (z)\quad (i = 1, \ldots ,n),$$

and at least for one *i*, the inequality is strict. In this case, z^*^ is also called a *cooperative solution of the n-person game* with strategy sets *D*_*i*_, payoff functions $f_{1} ,f_{2} , \ldots ,f_{n}$ and constraints (3)–(4). The set *P* of the above Pareto optimal function values *F*(z^*^) is called the *Pareto frontier of function* $F\left| {_{G} } \right.$(Blasco et al., 2008):

$$P = \left\{ {F(z)\left| {z \in G\;{\text{is}}\;{\text{Pareto}}\;{\text{optimal}}\;{\text{for}}\;\left. F \right|_{G} \, } \right.} \right\}$$

In principle, we can effectively find a large number Pareto optimal function values, by the following “scalarization” of the corresponding vector optimization problem:for every $\lambda = (\lambda_{1} ; \ldots ;\lambda_{n} ) \in \mathop \Delta \limits^{ \circ }_{n}$ (the relative interior of the standard simplex in **R**^*n*^, consisting of vectors with positive components summing 1), define the weighted sum $F_{\lambda } = \sum\limits_{i = 1}^{n} {\lambda_{i} f_{i} }$. Then, for any $\lambda \in \mathop \Delta \limits^{ \circ }_{n}$, any solution $z_{\lambda }^{ * }$ of the constrained scalar optimization problem

7 $$\mathop {F_{\lambda } (z)}\limits_{{z \in G}} \to \max$$

is Pareto optimal for the constrained vector optimization problem (6), and hence a cooperative solution of the considered game (Geoffrion, 1968).

##### ***Remark 1***

By giving equal weights to all players, with $\lambda = \left( {\frac{1}{n};...;\frac{1}{n}} \right)$, we obtain a cooperative solution providing a maximum total payoff to all players.

##### ***Remark 2***

Except for some degenerate cases, all points of the Pareto frontier can be obtained by solving the scalar optimization problem (7), for all $\lambda \in \mathop \Delta \limits^{ \circ }_{n}$ (Geoffrion, 1968). In this way, in general, we obtain an infinite set of cooperative solutions with different weighting for the *n* players. In what follows, we propose a particular way to select a single solution from this variety.

#### *Ideal value of the game*

Since set *G* of admissible multi-strategies is compact and the payoff functions $f_{i}$ are continuous, we can define $\omega_{i} = \mathop {\max }\limits_{z \in G} f_{i} (z)$ (*i* = 1,…,*n*), and $\Omega = (\omega_{1} ;\omega_{2} ; \ldots ;\omega_{n} )$. $\Omega$ can be called *the ideal value of the game*, since $\omega_{i}$ would be the best payoff for Player *i* but it is extremely rare that maxima $\omega_{i}$ are taken at the same *z* for all *i*. In other words, in general, $\Omega$ does not belong to the range of the vector-valued function $\left. F \right|_{G}$, but we can try to find a point $F(z^{0} ) \,$ on the Pareto frontier *P* of the restriction $\left. F \right|_{G}$, closest to the ideal value of the game:

$$d(\Omega ,P) = \mathop {\inf }\limits_{p \in P} \left| {\Omega - p} \right| \, = \left| {\Omega - F(z^{0} ) \, } \right| \,$$.

Now such multi-strategy $z^{0}$ will be called *nearly ideal cooperative solution* of the game (Salukvadze, 1974; Varga, 1978).

##### ***Remark 3***

We note that if the Pareto frontier *P* is a closed set, then the distance $d(\Omega ,P)$ is actually reached at a point $p \in P$. Nevertheless, the Pareto frontier is not necessarily closed, except the linear case (Greenberg, 2010). If *P* is closed and convex, the distance $d(\Omega ,P)$ is reached at a unique point of *P.* In the present application, the numerical realization will deal with a finite subset of the Pareto frontier, so the corresponding minimum distance will be obviously reached.

## Results

For an illustration of our general game-theoretical model (1)–(6) presented in the previous section, in the following examples, based on Tables 1 and 2, we consider the flow tube 3, which consists of seven players (thermal plants) of three different kinds (with different maximal flow rate values). In order to understand the roles of different kinds of players, we analyze three different game structures, that is, a game with two, three and seven players.

*Data of flow tube 3 (for seven players)*

*n* = 7

$$Qe\,\rm {min} _{1} = 2.27\,m^{3} /h,\quad \textit{Qe}\,\rm {max}_{1} = 8.86\,m^{3} /h$$

$$Qe\,\rm {min}_{2} = 2.27\,{\text{m}}^{3} /{\text{h}},\quad \textit{Qe}\,\rm {max}_{2} = 8.86\,{\text{m}}^{3} /{\text{h}}$$

$$Qe\,\rm {min}_{3} = 2.27\,{\text{m}}^{3} /{\text{h}},\quad \textit{Qe}\,\rm {max}_{3} = 8.86\,{\text{m}}^{3} /{\text{h}}$$

$$Qe\,\rm {min}_{4} = 2.27\,{\text{m}}^{3} /{\text{h}},\quad \textit{Qe}\,\rm {max}_{4} = 8.86\,{\text{m}}^{3} /{\text{h}}$$

$$Qe\,\rm {min}_{5} = 2.27\,{\text{m}}^{3} /{\text{h}},\quad \textit{Qe}\,\rm {max}_{5} = 23.62\,{\text{m}}^{3} /{\text{h}}$$

$$Qe\,\rm {min}_{6} = 2.27\,{\text{m}}^{3} /{\text{h}},\quad \textit{Qe}\,\rm {max}_{6} = 26.27\,{\text{m}}^{3} /{\text{h}}$$

$$Qe\,\rm {min}_{7} = 2.27\,{\text{m}}^{3} /{\text{h}},\quad \textit{Qe}\,\rm {max}_{7} = 26.27\,{\text{m}}^{3} /{\text{h}}$$

$$Q = 26.28\,{\text{m}}^{3} /{\text{h}},\quad V = 630.7\,{\text{m}}^{3}$$

$$p_{1}$$ = 80 €/m^3^ and $$p_{2}$$ = 5 €/m^3^.

With the above data the game model (1)–(5) has been considered.

The Matlab software (Etter et al., 2004) and its fmincon command was used to carry out the numerical calculations with respect to the constrained scalar optimization problem (7) in the below examples.

### ***Example 1***

Cooperative solution with maximum total payoff to the community of all players.

Based on Remark 1 of the previous section, with choice $\lambda = \left( {\frac{1}{7}; \ldots ;\frac{1}{7}} \right)$, we have to solve the scalar optimization problem

$$\mathop {F_{\lambda } (z)}\limits_{{z \in G}} \to \max$$

For the solution $z^{ * } = ((x_{1}^{ * } ;t_{1}^{ * } ;\tau_{1}^{ * } ); \ldots ;(x_{7}^{ * } ;t_{7}^{ * } ;\tau_{7}^{ * } ))$, we obtain

$$z^{ * } = \left( {3.75;6;18;3.75;6;18;3.75;6;18;3.75;6;18;3.75;6;18;3.75;6;18;3.75;6;18} \right).$$

This means that every unit has to use the same strategy:

$(x_{i}^{ * } ;t_{i}^{ * } ;\tau_{i}^{ * } ) = (3.75;6;18)\quad (i = 1, \ldots ,7)$,with maximal objective value.

$F_{\lambda } (z*) = \sum\limits_{i = 1}^{7} {\frac{1}{7}f_{i} } (x_{i}^{ * } ;t_{i}^{ * } ;\tau_{i}^{ * } ) = 2139.9$,and the total payoff (total daily income) is 7 × 2139.9 = 14,975 (€).

The above symmetric strategy choice of each player (with 3.75 m^3^/h, 6 h and 18 h) serves with the maximal sum of the payoffs of the separated players (maximal total payoff of the game) with the same 2139.9 € payoff for each player.

For what follows, we note that the geometric illustration of the Pareto frontier for more than three players is a rather complicated issue (Blasco et al., 2008). We solve this problem below, in part by considering certain “subgames” of lower dimension, in part by considering 2- and 3-dimensional projections of the original, 7-dimensional construction. $Q =$ 26.28 m^3^/h, $V =$ 630.72 m^3^, $p_{1}$ = 80 €/m^3^ and $p_{2}$ = 5 €/m^3^ are general parameter values of the examples below.

### ***Example 2***

Finding nearly ideal cooperative solution in case of two players.

Now *n* = 2. For Player 1, $Qe\min_{1}$ = 2.27 m^3^/h, $Qe\max_{1}$ = 8.86 m^3^/h. For Player 2, $Qe\min_{2}$ = 2.27 m^3^/h, $Qe\max_{2}$ = 23.62 m^3^/h.

First, the ideal value of the game is determined. Its *i*-th coordinate is $\omega_{i} = p_{1} \cdot Qe\max_{i} \cdot 6 + p_{2} \cdot Qe\max_{i} \cdot 18$.

Then the scalar optimization procedure is run for 9,999 $\lambda = \left( {\lambda_{1} ;\lambda_{2} } \right)$ vectors, based on uniform divisions of interval [0,1]. The nearly ideal cooperative solution (more precisely its approximation) is selected from the gained Pareto optimal points, according to the minimum distance from the ideal value (see Remark 3). The results are the following:

*Ideal value of the game*

$$\Omega \, = \,\left( {5047.9;13,461.1} \right)$$

*Nearly ideal cooperative solution*

Obtained for $$\lambda$$ = (0.5;0.5).

Corresponding multi-strategy $z^{0} = \left( {\left( {x_{1} ;t_{1} ;\tau_{1} } \right);\left( {x_{2} ;t_{2} ;\tau_{2} } \right)} \right) = \left( {\left( {7.26;6;18} \right);\left( {19.02;6;18} \right)} \right)$,

Payoff vector $$F\left( {z^{0} } \right) = \left( {4183.3;10,841.3} \right)$$.

**Fig. 3:**
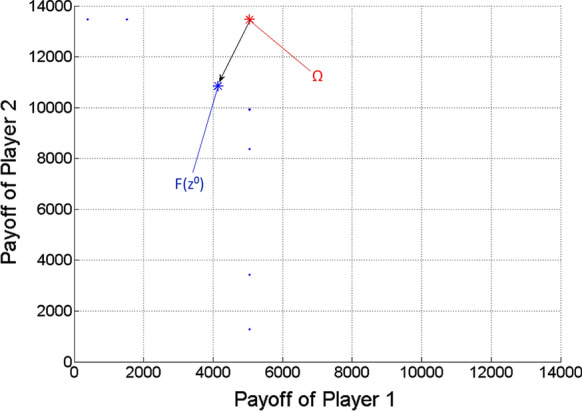
Pareto optimal payoff vectors/points and the ideal value in the two-player game

Figure 3 shows the gained Pareto optimal payoff vectors/points as blue dots, among which the one $\left( {F\left( {z^{0} } \right)} \right)$ with respect to the nearly ideal cooperative solution of the game ($z^{0}$) is highlighted as a large blue asterisk. The ideal value of the game is also highlighted as a large red asterisk. The black arrow indicates the projection of the ideal value to the Pareto frontier.

### ***Example 3***

Finding nearly ideal cooperative solution in case of three players.

Now *n* = 3. For Player 1, $Qe\min_{1}$ = 2.27 m^3^/h, $Qe\max_{1}$ = 8.86 m^3^/h. For Player 2, $Qe\min_{2}$ = 2.27 m^3^/h, $Qe\max_{2}$ = 23.62 m^3^/h. For Player 3, $Qe\min_{3}$ = 2.27 m^3^/h, $Qe\max_{3}$ = 26.27 m^3^/h.

After determining the ideal value of the game, based on uniform divisions of interval [0,1], the scalar optimization procedure is run for 19,486 $\lambda = \left( {\lambda_{1} ;\lambda_{2} ;\lambda_{3} } \right)$ vectors (or Pareto optimal points), from which the nearly ideal cooperative solution is selected according to the minimum distance from the ideal value. The results are the following:

*Ideal value of the game*

$$\Omega = \left( {5047.9;13,461.1;14,975.5} \right)$$

*Nearly ideal cooperative solution*

Obtained for $$\lambda = \left( {0.02;0.49;0.49} \right),$$

Multi-strategy $$z^{0}$$ = $$\left( {\left( {x_{1} ;t_{1} ;\tau_{1} } \right);\left( {x_{2} ;t_{2} ;\tau_{2} } \right);\left( {x_{3} ;t_{3} ;\tau_{3} } \right)} \right) = \left( {\left( {2.27;6;18} \right);\left( {10.56;6;18} \right);\left( {13.45;6;18} \right)} \right),$$

Payoff vector $$F\left( {z^{0} } \right) = \left( {1292.8;6020.3;7666.5} \right).$$

**Fig. 4:**
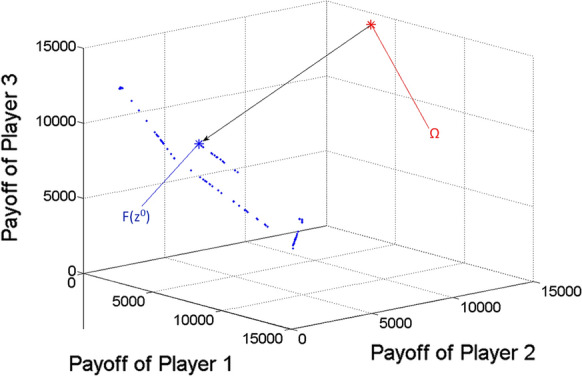
Pareto optimal payoff vectors/points and the ideal value in the three-player game

Figure 4 shows the gained Pareto optimal payoff vectors/points as blue dots, among which the one $\left( {F\left( {z^{0} } \right)} \right)$ with respect to the nearly ideal cooperative solution of the game ($z^{0}$) is highlighted as a large blue asterisk. The ideal value of the game is also highlighted as a large red asterisk. The black arrow indicates the projection of the ideal value to the Pareto frontier.

**Fig. 5:**
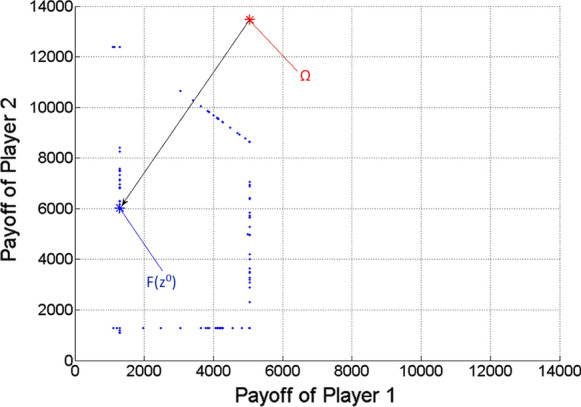
Pareto optimal payoff vectors/points and the ideal value in the three-player game, from the viewpoint of Players 1 and 2

**Fig. 6:**
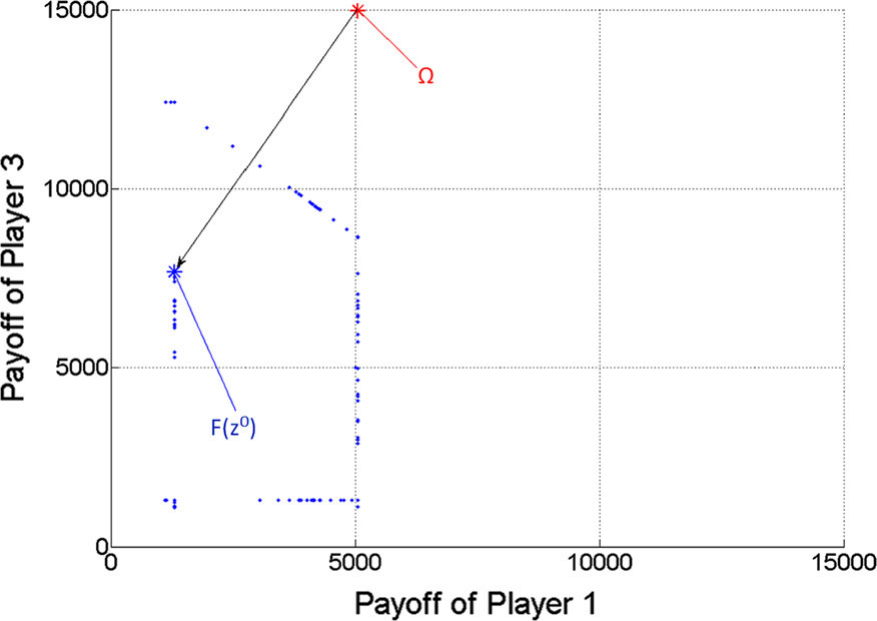
Pareto optimal payoff vectors/points and the ideal value in the three-player game, from the viewpoint of Players 1 and 3

**Fig. 7:**
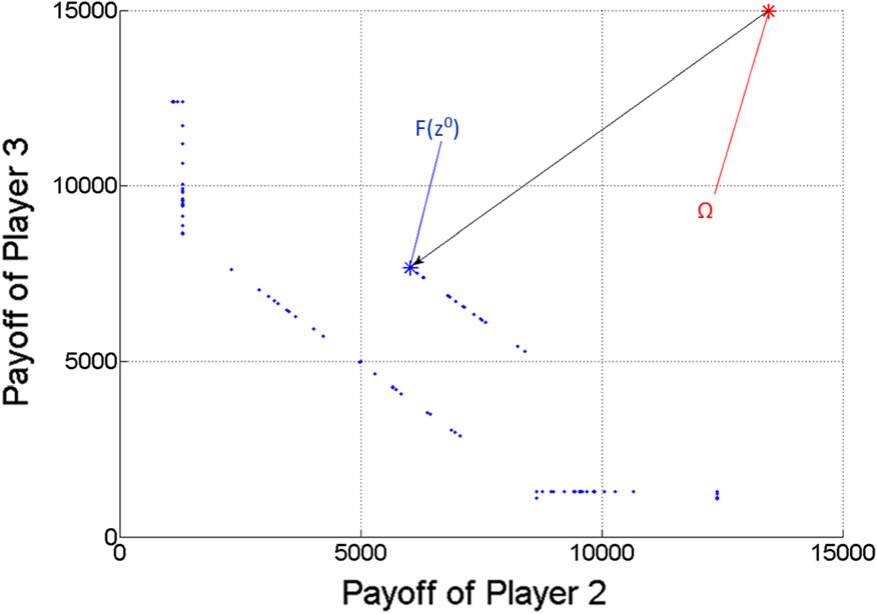
Pareto optimal payoff vectors/points and the ideal value in the three-player game, from the viewpoint of Players 2 and 3

Figures 5, 6 and 7 show the two-dimensional projections of Fig. 4 from the viewpoint of any two players from the three ones. Naturally, because of the missing third dimension, F(z^0^) must not seem to be the closest point to $\Omega$ among the Pareto optimal points (blue dots) in Figs. 5, 6 and 7.

### ***Example 4***

Finding nearly ideal cooperative solution in case of seven players.

The parameter values of the game are as given at the beginning of the present Section.

After determining the ideal value of the game, based on uniform divisions of interval [0,1], the scalar optimization procedure is run for 27,132 $\lambda = \left( {\lambda_{1} ;\lambda_{2} ; \ldots ;\lambda_{7} } \right)$ vectors (or Pareto optimal points), from which the nearly ideal cooperative solution is selected according to the minimum distance from the ideal value. The results are the following:

*Ideal value of the game*

$$\Omega = \left( {5047.9;5047.9;5047.9;5047.9;13,461.1;14,975.5;14,975.5} \right)$$

*Nearly ideal cooperative solution*

Obtained for $$\lambda = \left( {0.1;0.1;0.1;0.1;0.2;0.2;0.2} \right),$$

Multi-strategy $$\begin{aligned} z^{0} & = \left( {\left( {x_{1} ;t_{1} ;\tau_{1} } \right);\left( {x_{2} ;t_{2} ;\tau_{2} } \right); \ldots ;\left( {x_{7} ;t_{7} ;\tau_{7} } \right)} \right) \\ & = \left( {\left( {2.268;6;18} \right);\left( {2.268;6;18} \right);\left( {2.268;6;18} \right);\left( {2.268;6;18} \right);\left( {7.273;6;18} \right);\left( {4.968;6;18} \right);\left( {4.968;6;18} \right)} \right), \\ \end{aligned}$$.

Payoff vector $$F\left( {z^{0} } \right) = \left( {1292.8;1292.8;1292.8;1292.8;4145.6;2831.5;2831.5} \right).$$

**Fig. 8:**
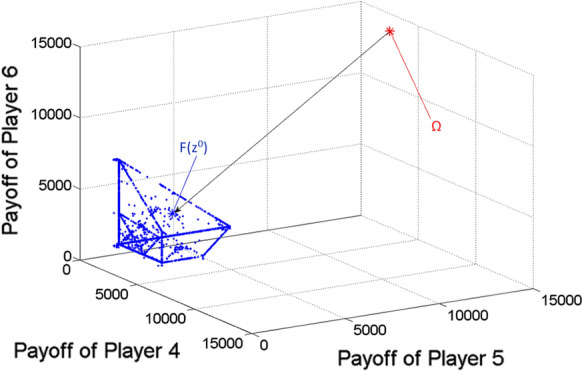
Pareto optimal payoff vectors/points and the ideal value in the seven-player game, from the viewpoint of Players 4, 5 and 6

From the viewpoint of the three different kinds of players (namely Players 4, 5 and 6), Fig. 8 shows the three-dimensional projection of the gained Pareto optimal payoff vectors/points as blue dots and the ideal value of the game as a large red asterisk. The black arrow indicates the projection of the ideal value to the Pareto frontier. Among the Pareto optimal payoff vectors, the one $\left( {F\left( {z^{0} } \right)} \right)$ with respect to the nearly ideal cooperative solution of the game ($z^{0}$) is highlighted as a large blue asterisk. Naturally, because of the missing four dimensions, $F\left( {z^{0} } \right)$ may not seem to be the closest point to $\Omega$ among the Pareto optimal points (blue dots) in the figure.

**Fig. 9:**
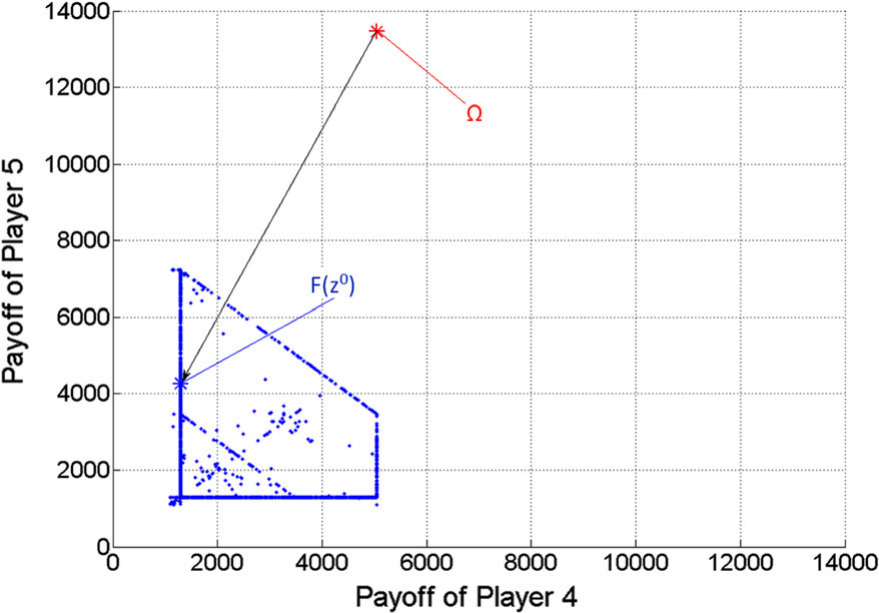
Pareto optimal payoff vectors/points and the ideal value in the seven-player game, from the viewpoint of Players 4 and 5

**Fig. 10:**
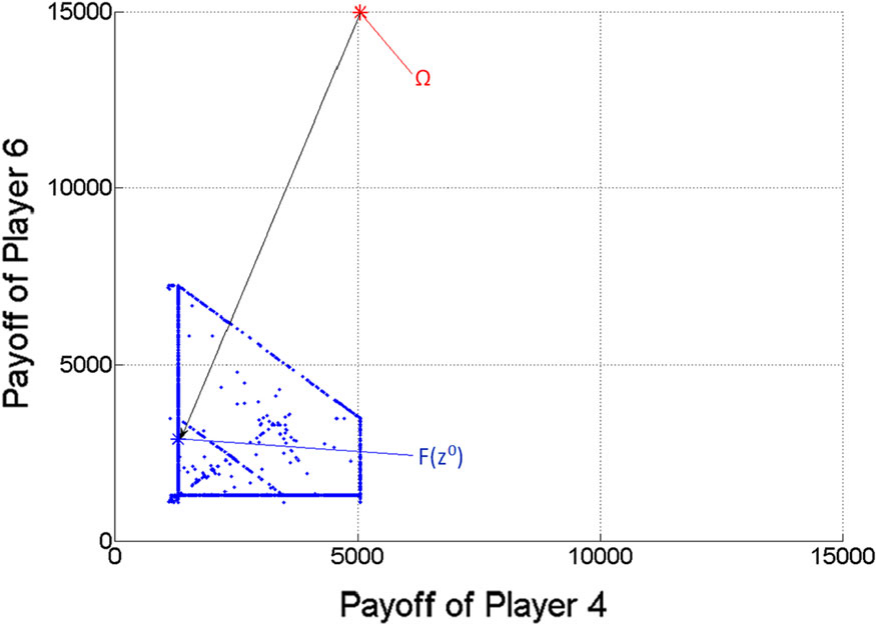
Pareto optimal payoff vectors/points and the ideal value in the seven-player game, from the viewpoint of Players 4 and 6

**Fig. 11:**
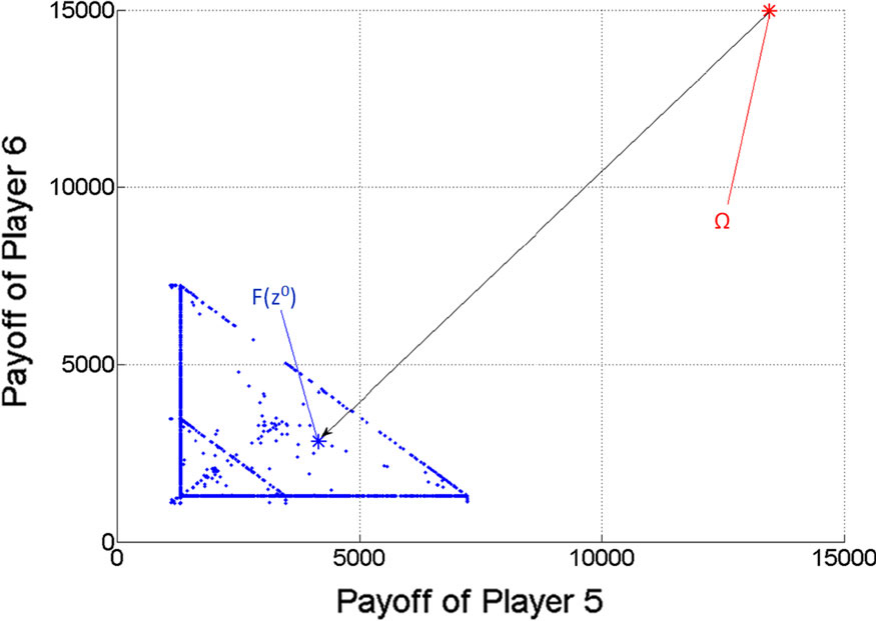
Pareto optimal payoff vectors/points and the ideal value in the seven-player game, from the viewpoint of Players 5 and 6

Figures 9, 10 and 11 show the two-dimensional projections of Fig. 8 from the viewpoint of any two players from the mentioned three ones (from Players 4, 5 and 6). Naturally, because of the missing five dimensions, $F\left( {z^{0} } \right)$ may not seem to be the closest point to $\Omega$ among the Pareto optimal points (blue dots) in Figs. 9, 10 and 11.

### ***Remark 4***

We note that relatively few points (blue dots) can be seen in Figs. 3–11, in comparison with the relatively large number of $\lambda$ vectors (9,999, 19,486 and 27,132). The reason must be that the used scalar optimization procedure results in the same Pareto optimal point for many different lambda vectors.

### ***Remark 5***

In Tables 3 and 4, for short we call Solution A that maximizes the total daily income (Example 1), and Solution B is the nearly ideal cooperative solution (Example 4), in both cases for flow tube 3.

**Table 3:** Fulfillment of sustainability conditions for Solutions A and B for the flow tube 3

Plant	Solution A		Solution B	
	VP (m^3^)	VF (m^3^)	VP (m^3^)	VF (m^3^)
5	67.5	22.5	40.824	13.608
6	67.5	22.5	40.824	13.608
7	67.5	22.5	40.824	13.608
9	67.5	22.5	40.824	13.608
17	67.5	22.5	130.914	43.638
27	67.5	22.5	89.424	29.808
36	67.5	22.5	89.424	29.808
Total flow	472.5	157.5	473.058	157.686
	VP + VF = 630		VP + VF = 630.744

**Table 4:** Comparison of current daily incomes of plants (units) and the incomes resulting from the game model for the flow tube 3

Plant	Current daily income (€)	Daily income (€) Solution A	Daily income (€) Solution B
5	3700	2139	1292.8
6	2530	2139	1292.8
7	5030	2139	1292.8
9	3650	2139	1292.8
17	3650	2139	4145.6
27	3310	2139	2831.5
36	2210	2139	2831.5
Total income	24,080	14,973	14,979.8

In Table 3, for both Solutions A and B, the volume limit is analyzed. The prescribed sustainability limit for the total daily volume was $V =$ 630.72 m^3^, and, as we can see in both solutions, the available resource (up to roundup error) is exhausted. Similar result is easily obtained for the total pumping flow rate: constraint (4) is fulfilled as equality.

In Table 4, sustainable solutions are compared to the current practice in economic terms. First of all, in both Solutions A and B (obtained in Examples 1 and 4, respectively) the total income is substantially reduced by the sustainability constraints. In Solution A, the reduced total income is uniformly distributed among plants. In Solution B, from the same available total daily income, the plants with higher capacity (maximum flow rate of pumping, *Qe*_max_) would earn even more than their current income. The all over decrease in daily income in Solution A, with respect to the current daily income, is obviously the cost of sustainability.

Finally, we emphasize that letting run the vector $\lambda$ of weights, in principle, an infinite variety of cooperative solutions can be obtained, among them one can decide according to a further criterion.

## Discussion

The mathematical model developed through the theory of games has been addressed to verify possible solutions for a sustainable use of hydrothermal resources of the Island of Ischia. On the island, there is a considerable concentration of groundwater extraction from wells, especially in the coastal areas, for the supply of the many spas on which the local economy depends. The absence of a regulatory plan for groundwater withdrawals may give rise to variations in the quality of the thermal waters used for therapeutic purposes during the peak of the tourist season (Piscopo et al., 2020a, b). The model has been implemented to resolve the conflict between the different users of hydrothermal resources who insist on the same portion of the aquifer, considering the main hydrogeological constraints to contain the variations of the quality of the water pumped from wells and the maximum profit to the users of the resource. The model was applied to one of the sectors of the island where critical conditions between yield of the groundwater resources and withdrawals exist.

The results of the model in the specific critical sector of the island were summarized in terms of flow rates and volumes of water that can be extracted by individual users and related income (Tables 3 and 4). The different possible solutions, synthesized in those called Solution A and Solution B, that respect the hydrogeological constraints for maintaining the quality of the water extracted from the wells over time, imply a reduction in the quantity of pumping from the single well.

Solution A, which implies an equal distribution of resources among the different users, gives rise to a percentage reduction in the daily volumes for spa treatments (VF) not exceeding 45%; for two plants no reduction in VF occurs. This has the most influence on the reduction in the daily income of the single plant. All plants, on the other hand, suffer a reduction in the daily volumes for the filling of the pools (VP), which has a lower impact on total daily income.

Solution B, which implies an unequal distribution of resources among the different users in relation to local hydrogeological constraints, gives rise to a higher percentage reduction in VF (up to 67%) for the plants closest to the coast. This is due to the low initial elevation of the groundwater level ASL and the restraint of the drawdown induced by pumping. Instead, the wells of the island’s innermost plants could also increase the volumes of the pumped water, therefore increasing their daily income (Tables 2, 3 and 4). Even for this solution, a reduction in VP results. While the thermal waters for spa treatments (i.e., VF) must necessarily be pumped daily by users, an alternative plan for filling pools can be found by distributing the VP withdrawal over more days.

We note that we have presented our game-theoretical approach to sustainable management only for flow tube 3, as an illustration. Taking into consideration the particular features of the corresponding datasets, similar game-theoretical models can be applied to the rest of the five flow tubes of the area under examination or the other areas of the island. In addition, the solutions resulting from the model only consider the hydrogeological and economic aspects of the problem, assuming the current location and depth of wells as constraints. Instead, what results from Solution B suggests that new scenarios for sustainable resource management including, for example, the repositioning of wells within the flow tube (one of the factors that affect the containment of the change in water quality during pumping) may be possible.

It should also be noted that the hydrogeological constraints, adopted on the basis of the containment of the variation in the quality of water pumped from wells, relate to a simplification of the distribution of the hydraulic head, the transmissivity of the aquifer and the specific capacity of wells within the flow tube. More detailed knowledge of the distribution of these parameters within the flow tube can be included in the model, resulting in a variation of coordinate *x*_*i*_ of the model.

In more general terms, the developed model indicates that for *i* = 1,…,*n*, player *i*’s payoff (2) depends directly only on the coordinates of its strategy $(x_{i} ;t_{i} ;\tau_{i} )$ in a strictly monotonically increasing way. This means that for any Pareto optimal multi-strategy, $x_{i}$ = $Qe\max_{i}$, $t_{i}$ = 6 h, $\tau_{i}$ = 18 h (*i* = 1,…,*n*) and/or $\sum\limits_{i}^{n} {x_{i} } = Q$ (see constraint (3)) and/or $\sum\limits_{i}^{n} {x_{i} } t_{i} + \sum\limits_{i}^{n} {x_{i} } \tau_{i} = V$(see constraint (4)) hold, otherwise, at least one of the players’ strategies (more precisely, either $x_{i}$, $t_{i}$ or $\tau_{i}$ for some *i* = 1,…,n) could be improved (increased) to increase the corresponding player’s payoff, without decreasing others’ payoffs. That is, the considered multi-strategy would not be really Pareto optimal. It also follows from this that each Pareto optimal multi-strategy is also a Nash equilibrium of the game, since with respect to a Pareto optimal point, no player can increase any coordinate of its strategy vector (according to the above strategy maximizing equalities) and consequently, its payoff value. A multi-strategy is called a *Nash equilibrium, or non-cooperative solution* of the game if neither player can deviate unilaterally from this multi-strategy (while the others do not deviate from it) to increase its own payoff (regardless of the others’ payoffs).

In a further development of the present work, the authority responsible for the management and safeguarding of the hydrothermal resources can be included as an additional actor, in the role of a distinguished player which adjusts other constraints in the use of resources. Furthermore, this authority may have priority in time before the other players (the thermal plants), which leads us to the special game-theoretical field of Stackelberg (or leader–follower) games. Such type of games and their solutions with connections to water resource management can be found in Kicsiny et al. (2014a) and Kicsiny (2017). Additionally, it may be worth working out the dynamic version of the proposed game taking into account the (interconnected) effects of the time-dependency of the players’ consumptions, similarly as in the differential game model of Kicsiny and Varga (2019).

## Conclusions

This study proposes a new approach to managing groundwater pumping from the volcanic aquifer of the Island of Ischia, an active hydrothermal system where numerous spas extract thermal waters that are significantly different in temperature, salinity and chemical compositions. A game-theoretical modeling was implemented to obtain the optimal sustainable exploitation of the groundwater resources, in order to maintain the quality of thermal waters over time and maximize the profit of the different competing users of the resource (spas) falling in the same flow tube of the aquifer. In the game the spas are the players, the strategy of a player consists of a fixed pumping rate and daily time durations of pumping, and the player’s utility or payoff is proportional to the total quantity of withdrawn thermal water in a given time period. A special constrained Pareto optimal strategy choice is obtained, considered as a cooperative solution of the game. Pareto optimality means that there is no other strategy choice that makes one player better off without making some other player worse off.

Although the model has been tested only one flow tube of one of the areas with the highest density of island’s wells, it is evident that future management of the island’s hydrothermal resources requires a new approach based on local hydrogeological conditions (mainly elevation of the groundwater level and drawdown induced by pumping) and on the balance between natural flow and quantity of volumes extracted from the single flow tube. As for the latter point, at least, participatory management in the use of this important hydrothermal resource, on which the economy of the island directly and indirectly depends, is necessary. The mathematical model implemented can represent a useful tool for this purpose, as well as for other hydrothermal systems, where typically quality and quantity of water extracted determine the economic return of spas.

Finally, as for the novelty of our mathematical modeling approach, we note that Pareto optimality has already been widely used in conflict situations like resource allocation, a very recent overview on that is Null et al. (2020). However, the main part of our game-theoretical modeling, the application of the *nearly ideal cooperative solution* in water resource management, seems to be a promising new solution for further applications to more complex resource allocation problems.

## Data Availability

The datasets generated and/or analyzed during the current study are available from the corresponding author on reasonable request.
